# Correlation between bone turnover and metabolic markers with age and gender: a cross-sectional study of hospital information system data

**DOI:** 10.1186/s12891-020-03610-w

**Published:** 2020-09-10

**Authors:** Ju Shao, Shao-Song Zhou, Yuan Qu, Bi-Bo Liang, Qing-Hong Yu, Jing Wu

**Affiliations:** 1 Department of Rheumatology and Clinical Immunology, ZhuJiang Hospital, Southern Medical University, 253 Gongye Ave, Guangzhou, 510282 Guangdong China; 2 Department of Laboratory, ZhuJiang Hospital, Southern Medical University, Guangzhou, China

**Keywords:** Bone turnover, Bone metabolic, Gender, Age, Children

## Abstract

**Background:**

Bone turnover and metabolic indicators are related to age and gender. Age and gender should be matched in subjects in disease control research of bone turnover and metabolism, but strict matching of gender and age increases the difficulty and cost of the research. Therefore, the aim of this study was to solve it is necessary to strictly match age and gender in clinical research in bone metabolism.

**Methods:**

A cross-sectional study was conducted from the data were extracted from the HIS of ZhuJiang Hospital. Data relating to seven bone turnover and metabolic indicators from 1036 patients between January 2018 and October 2019 were analyzed.

**Results:**

P1NP, β-CTx and 25(OH)D were significant different in individuals younger than 20 years of age. ALP was significantly higher in those under 20 years of age and lower at age 20–39 compared with other age groups. The concentrations of Ca and P were different among the groups aged 0–19, 20–39, and 40–59 years of age groups but exhibited no difference above 60 years of age. PTH expression was not dependent on age. P1NP, β-CTx and PTH concentrations were not significantly different between the genders within the same age group. ALP was significantly different between genders within the age range 20–59 years. Ca and 25(OH)D were significantly different between the genders for those older than 60. Serum P was significantly different in the two genders for those aged 40–79. Patients received both alfacalcidol and calcium treatment differently from the others in P1NP, β-CTx, Serum Ca, P and ALP.

**Conclusion:**

P1NP and β-CTx were highly correlated with age. If these two indictors require analysis in a case control study, the patients and controls should be strictly matched by age under 20 years. The demarcation point for ALP was 40 years of age. Ca and P were strongly recommended strict matching according to age in disease research. The difference in P1NP, β-CTx, 25(OH)D and ALP between genders depends on age differences. Medication history should be considered in bone turnover and metabolic clinical research.

## Background

The skeletal system operates at different levels of metabolic activity in different disease states which also varies with age [1]. Bone metabolism indicators have been studied well in detail menopausal women, children and adolescents. The majority of researchers believe that the age and gender of subjects should be matched in bone metabolism research. It remains unclear whether research of the control of bone metabolic diseases requires the strict matching of the age and sex of patients. Also unclear is which is more important to strictly match, age or gender. Strict matching involves great difficulty and high costs, especially in randomized controlled trials (RCTs). It is unclear whether strict matching is in fact required at every age. Since no epidemiological research is able currently to answer this question, especially that of infants and very young children aged 0–5 years old, we wished to investigate such relevant information from inpatient data as a reference. We suppose that there is association between markers of bone formation or turnover with age and gender. Therefore, the aim of this study was to answer the question of matching. We assessed the relationship between markers of bone formation or turnover with age and gender, fitted the most appropriate curve and inferred the apparent relationship between them. Thus, data for seven bone indicators related to bone formation, turnover and metabolism were extracted, and their association with age and gender analyzed. The markers measured were amino-terminal propeptide of type I collagen (P1NP), beta-isomerized C-terminal telopeptide of type I collagen (β-CTx), alkaline phosphatase (ALP), 25-hydroxyvitamin D (25(OH)D), parathyroid hormone (PTH), calcium (Ca) and phosphorus (P) in serum.

## Methods

### Study design

A cross-sectional study was conducted to assess the association between bone turnover and metabolic indicators with age and gender in inpatients. Because this study focused on age matching for disease studies, all patient data were extracted from the hospital information system (HIS) of ZhuJiang Hospital, Southern Medical University, from January 2018 to October 2019. Seven indicators from patients were retrieved. P1NP, β-CTx, 25(OH)D and PTH were analyzed at ZhuJiang Hospital, Guangzhou, China, using the method of ECLIA (Electro chemiluminescent immunoassay), and ALP was measured by the rapid colorimetric test. Serum calcium and P were measured with the colorimetric kit, according to the manufacturer’s guidelines. No additional medical procedures were performed on patients as all medical record data of patients who had already received treatment had already been recorded and stored within the computer system. ZhuJiang Hospital of Southern Medical University Ethics Committee waived the approval due to the retrospective nature of the study.

### Patients

We selected as many patients as possible from the (HIS) of ZhuJiang Hospital, Southern Medical University. From January 2018 to October 2019, the records of 1036 subjects in the age range from infant to 90 years who had undergone a biochemical indicator test of bone turnover and metabolism in ZhuJiang Hospital of Southern Medical University were obtained. The levels of bone metabolism are different in age change, so all patients were grouped by age to hierarchically analyze the bone indicators of different age brackets, including 0–19,20-39,40-59,60–79 and over 80 years of age. A total of 156 patients aged under 20 were identified and analyzed separately. Levels of P1NP, β-CTx, ALP, 25(OH)D, PTH, Ca and P in serum had been recorded in each case during routine laboratory investigation. Kinds of disease of all the patient’s data in supplement table 1. To reduce information bias, the data were collected by four experienced researchers, who divided into two teams, one team collected data and the other team checked the data.

### Statistical analysis

Statistical analysis was performed using IBM SPSS v24.0 (USA) software. Data are presented as median values (QR). Curve fitting and hierarchical analysis were conducted to quantify associations among the variables. Age-related changes in bone turnover and metabolic markers were modeled using third-degree polynomial functions, which gave the highest coefficients of determination (R^2^) in comparison with other models. Cubic and exponential curves were plotted from the calculated results. To determine potential factors for bone turnover and metabolic markers, multivariable logistic regression analysis was performed, and the associations between influence factors and outcomes are presented as odds ratios (ORs) and 95% CIs, after adjustment for confounders, including age, gender, types of anti-osteoporosis medicines, anti-osteoporosis medicines taking duration time, types of disease and bone turnover and metabolic detection season. Comparisons between groups were calculated using Chi-square, Mann-Whitney and Kruskal-Wallis tests. Use Max Likelihood analysis where missing data were taken into account. All statistical tests and confidence intervals were two-sided. *P* < 0.05 was considered statistically significant.

## Results

For all patients, the seven bone turnover and metabolic markers namely P1NP, β-CTx, ALP, Ca, P, 25(OH)D and PTH were extracted then analyzed. All baseline characteristics are presented in Table 1.

**Table 1:** Bone turnover and metabolic indicators are displayed hierarchically by age

Age phase (year)	0 ~ 19y	20 ~ 39y	40 ~ 69y	60 ~ 79y	80y~	***P*** value
**Gender**						
**Male(n)**	89	62	159	104	8	0.000
**Female(n)**	61	115	246	158	34	0.000
**P1NP (μg/L)**	520.05(340.90,825.28)	44.18(28.65,71.47)	44.59 (31.93,69.42)	44.25(29.66,62.45)	41.90(31.15,74.64)	0.000
**β-CTx (μg/L)**	1.35(1.01,1.73)	0.47(0.30,0.83)	0.50(0.30,0.75)	0.56(0.37,0.76)	0.42(0.28,0.78)	0.000
**PTH (pmol/L)**	3.05(2.20,4.20)	3.40(2.00,4.80)	3.30(2.47,4.83)	3.50(2.48,5.60)	3.25(2.27,5.60)	0.243
**Ca (mmol/L)**	2.53(2.43,2.64)	2.42(2.32,2.51)	2.41(2.32,2.51)	2.37(2.27,2.47)	2.30(2.21,2.42)	0.000
**P (mmol/L)**	1.56(1.40,1.75)	1.13(1.00,1.31)	1.12(1.00,1.27)	1.11(0.96,1.23)	1.12(0.90,1.21),	0.000
**25(OH)D(μg/L)**	23.80(18.75,29.20)	22.60(18.35,26.90)	22.40(18.00,27.20)	21.75(16.48,26.55)	19.20(14.70,26.38)	0.005
**ALP (mmol/L)**	221.50(148.98,279.25)	69.00(54.00,82.50)	78.50(65.00,103.00)	80.00(64.00,99.25)	83.43(63.25,99.00)	0.000

Data are presented as Median (QR). Statistical tests were made by Chi-square test and Kruskal-Wallis test. *P*-value among five age groups

### Correlation with age

**Fig. 1:**
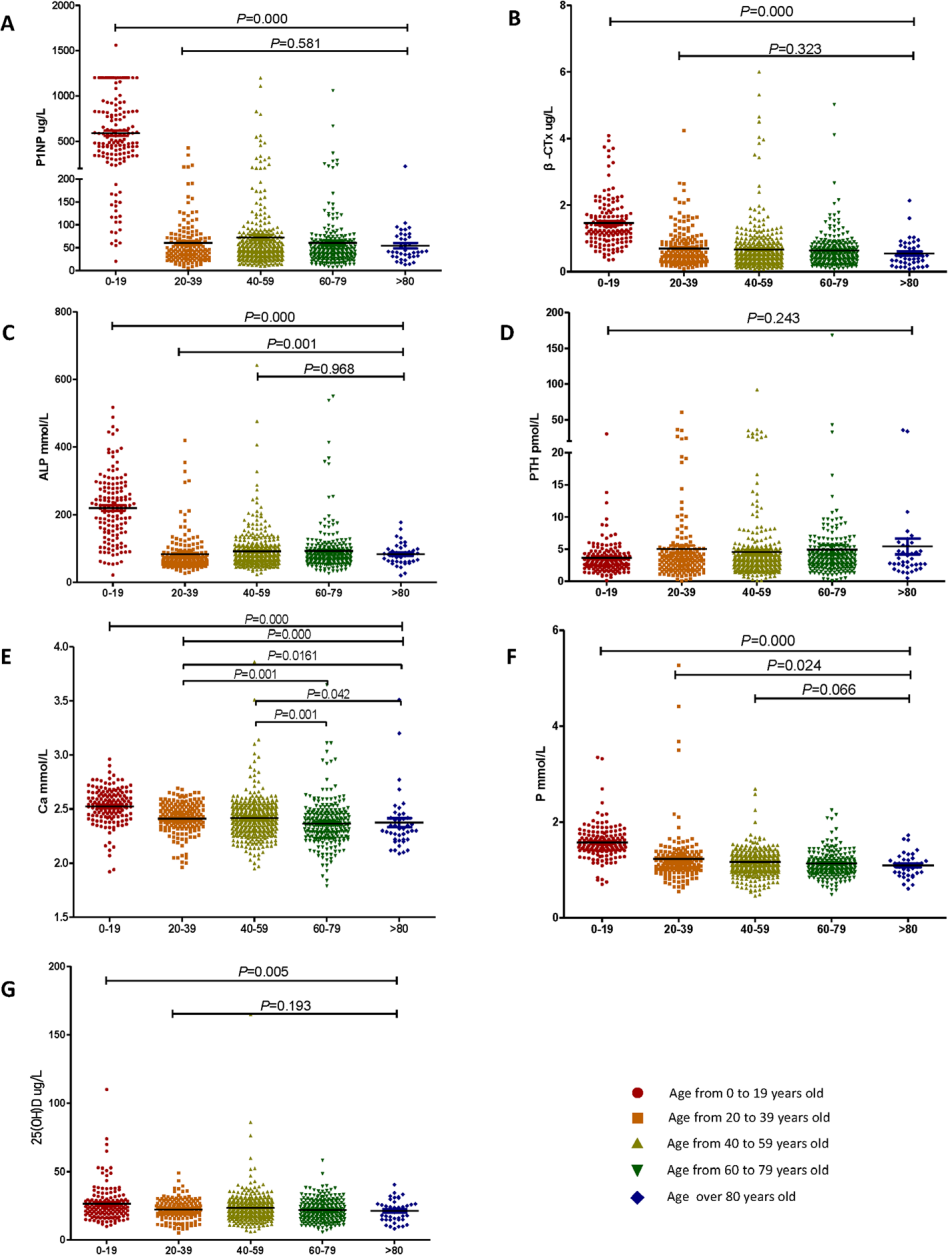
**a** Different expression of serum P1NP concentration in different ages; **b** Different expression of serum β-CTx concentration in different ages; **c** Different expression of serum ALP concentration in different ages; **d** Different expression of serum PTH concentration in different ages; **e** Different expression of serum Ca concentration in different ages; **f** Different expression of serum P concentration in different ages; **g** Different expression of serum 25(OH)D concentration in different ages; *p* < 0.05 as assessed by Mann–Whitney U test

Young patients had high levels of P1NP, β-CTx and 25(OH)D, the difference in their concentration in patients younger than 20 years compared with those who were older was significant, but not significant between groups for those over 20 years of age (Fig. 1a, b and g; Table 1). There was a significant difference in ALP concentrations for patients older than 20 years compared with those younger than 20 years age. ALP was significantly lower in patients aged 20–39 compared with other age groups. ALP concentration did not vary in patient groups over the age of 40 years (Fig. 1c; Table 1). The concentrations of Ca and P in different age groups were completely inconsistent, with differences among the 0–19, 20–39, and 40–59 years age groups, but not different over 60 years of age (Fig. 1e, f; Table 1). PTH expression was not dependent on age (Fig. 1d; Table 1).

**Fig. 2:**
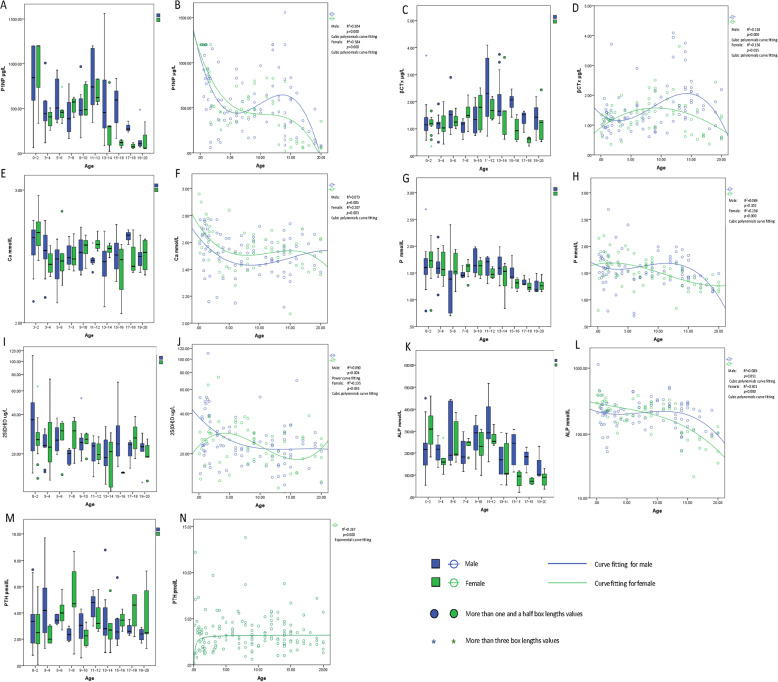
B Box plots of serum P1NP concentration by age and gender in patients under 20 years; **b** Cubic polynomial curves of serum P1NP concentration by age and gender in patients under 20 years; **c** Box plots of serum β-CTx concentration by age and gender in patients under 20 years; **d** Cubic polynomial curves of serum β-CTx concentration by age and gender in patients under 20 years; **e** Box plots of serum Ca concentration by age and gender in patients under 20 years; **f** Cubic polynomial curves of serum Ca concentration by age and gender in patients under 20 years; **g** Box plots of serum P concentration by age and gender in patients under 20 years; **h** Cubic polynomial curves of serum P concentration by age and gender in patients under 20 years; **i** Box plots of serum 25(OH)D concentration by age and gender in patients under 20 years; **j** Cubic polynomial curves of serum 25(OH)D concentration by age and gender in patients under 20 years; **k** Box plots of serum ALP concentration by age and gender in patients under 20 years; **l** Cubic polynomial curves of serum ALP concentration by age and gender in patients under 20 years; **m** Box plots of serum PTH concentration by age and gender in patients under 20 years; **n** Exponential curve of serum PTH concentration by age in female patients under 20 years; *p* < 0.05 as assessed by Regression analysis

**Fig. 3:**
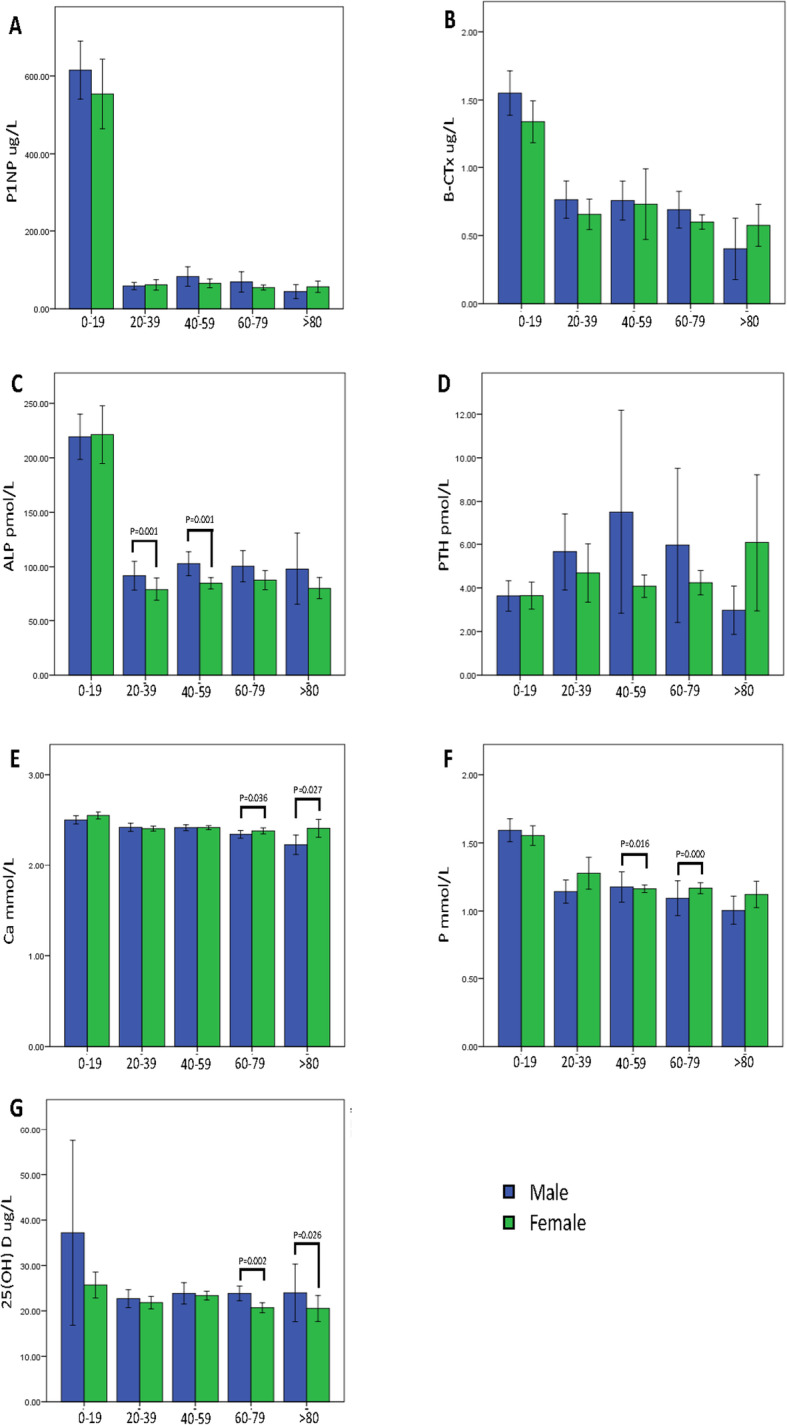
**a** Different expression of serum P1NP concentration in different ages and genders; **b** Different expression of serum β-CTx concentration in different ages and genders; **c** Different expression of serum ALP concentration in different ages and genders; **d** Different expression of serum PTH concentration in different ages and genders; **e** Different expression of serum Ca concentration in different ages and genders; **f** Different expression of serum P concentration in different ages and genders; **g** Different expression of serum 25(OH)D concentration in different ages and genders; *p* < 0.05 as assessed by Mann–Whitney U and Kruskal-Wallis tests

Because bone turnover and metabolic indicators were significantly different for those under 20 years of age, we analyzed and modeled those patients separately. In a total of 156 young patients, the relationship between P1NP and age was not a simple linear correlation. Regression analysis and curve fitting suggested that the relationship between P1NP and age was cubic polynomial. P1NP decreased with age below the age of 3–4, then increased over the range 4–14 years. Over14 year of age, P1NP then again declined with increasing age (Fig. 2a, b). β-CTx and ALP exhibited trends similar to those of P1NP, levels declining before the age of 4, then gradually increasing from 4 to 14 years, and gradually decreasing after 14 years until reaching the levels observed in adults (Fig. 3c, d, k, l). Serum Ca and P levels also fitted a cubic polynomial curve, although R^2^ was not greater than 0.3. (Fig. 2e-h). PTH and 25(OH)D levels did not exhibit a similar declining model but had a small rising trend with age (Fig. 2i-l).

### Correlation with gender

A stratified analysis was conducted according to the different age groups. The results indicated that serum P1NP, β-CTx and PTH were not significantly different between the two genders (Table 2, Fig. 3a, b and d). Serum ALP levels were significantly different for different genders for those aged 20–59 (Table 2, Fig. 3c). Serum Ca and 25(OH)D were significantly different in the two genders for patients older than 60 (Table 2, Fig. 3e, g). Serum P was significantly different in the two genders for those aged of 40–79 (Table 2, Fig. 3f). We additionally analyzed the bone markers of boys and girls aged between 0 to 19 years old. The age cut-off points for pre-pubertal and pubertal of boys are 12 years old and girls are 11 years old. We found only the levels of β-CTx (*p* = 0.006, Bonferroni Correction) has significant differences between pubertal boys and girls.

**Table 2:** Bone turnover and metabolic indicators are displayed hierarchically by gender and age

cAge phase (Year)	P1NP (μg/L)			β-CTx (μg/L)			PTH (pmol/L)			Ca (mmol/L)			P (mmol/L)			25(OH)D (μg/L)			ALP (mmol/L)		
	Male	Female	*P* value	Male	Female	*P* value	Male	Female	*P* value	Male	Female	*P* value	Male	Female	*P* value	Male	Female	*P* value	Male	Female	*P* value
0 ~ 19	575.90(341.80,833.15)	509.60(324.75,792.30)	0.333	1.39(1.05,1.82)	1.28(0.90,1.66)	0.167	3.15(2.20,3.98)	3.00(2.18,4.45)	0.813	2.53(2.43,2.64)	2.56(2.44,2.63)	0.400	1.54(1.40,1.74)	1.61(1.40,1.74)	0.937	25.15(19.03,29.45)	25.40(18.13,29.01)	0.955	216.00(148.94,277.50)	238.50(156.00,292.00)	0.591
20 ~ 39	52.09(30.31,73.23)	42.38(27.10,71.26)	0.134	0.93(0.62,1.41)	0.70(0.42,1.10)	0.058	3.90(2.80,4.85)	3.20(1.90,4.60)	0.067	2.44(2.33,2.54)	2.41(2.34,2.49)	0.297	1.10(0.97,1.27)	1.15(1.00,1.31)	0.119	21.15(18.43,27.00)	22.70(18.10,26.90)	0.553	80.00(64.75,97.50)	63.00(51.00.00,89.75.00)	0.001
40 ~ 69	43.04(31.03,64.26)	47.07(33.31,72.69)	0.517	0.51(0.33,0.72)	0.50(0.29,0.79)	0.526	3.30(2.40,4.65)	3.30(2.40,4.30)	0.975	2.41(2.32,2.50)	2.42(2.33,2.51)	0.705	1.08(0.97,1.27)	1.15(1.01,1.27)	0.016	21.00(17.20,27.95)	22.90(18.20,27.20)	0.392	84.00(69.00,111.00)	75.00(61.00,97.00)	0.001
60 ~ 79	38.37(27.49,56.79)	46.25(31.45,64.55)	0.099	0.54(0.36,0.74)	0.57(0.37,0.77)	0.624	3.40(2.40,5.25)	3.65(2.50,4.80)	0.653	2.35(2.23,2.44)	2.38(2.29,2.48)	0.036	1.00(0.89,1.13)	1.13(1.01,1.23)	0.000	23.00(17.55,29.23)	20.60(15.53,25.08)	0.002	85.00(65.75,105.25)	79.00(63.25,97.75)	0.283
80~	40.24(30.16,65.30)	43.57(31.15,77.88)	0.478	0.33(0.19,0.67)	0.48(0.31,0.85)	0.329	2.80(1.83,3.58)	4.15(2.28,6.08)	0.216	2.22(2.12,2.3)	2.34(2.22,2.44)	0.027	0.96(0.90,1.13)	1.13(0.91,1.32)	0.182	26.10(18.20,30.48)	17.7(14.63,25.45)	0.026	85.50(71.59,125.5)	82.43(63.00,99.00)	0.298
total	52.22(33.65,162.75)	48.76(33.55,80.15)	0.007	0.63(0.41,1.13)	0.52(0.33,0.87)	0.000	3.40(2.40,4.60)	3.30(2.30,4.60)	0.983	2.42(2.31,2.53)	2.41(2.32,2.52)	0.653	1.13(0.97,1.42)	1.16(1.02,1.33)	0.087	22.50(17.70,28.65)	22.35(17.40,26.80)	0.036	93.00(70.00,148.96)	77.00(61.00,101.85)	0.000

Data are presented as Median (QR). Statistical test was made by Mann-Whitney test

### Correlation with anti-osteoporosis medication

**Fig. 4:**
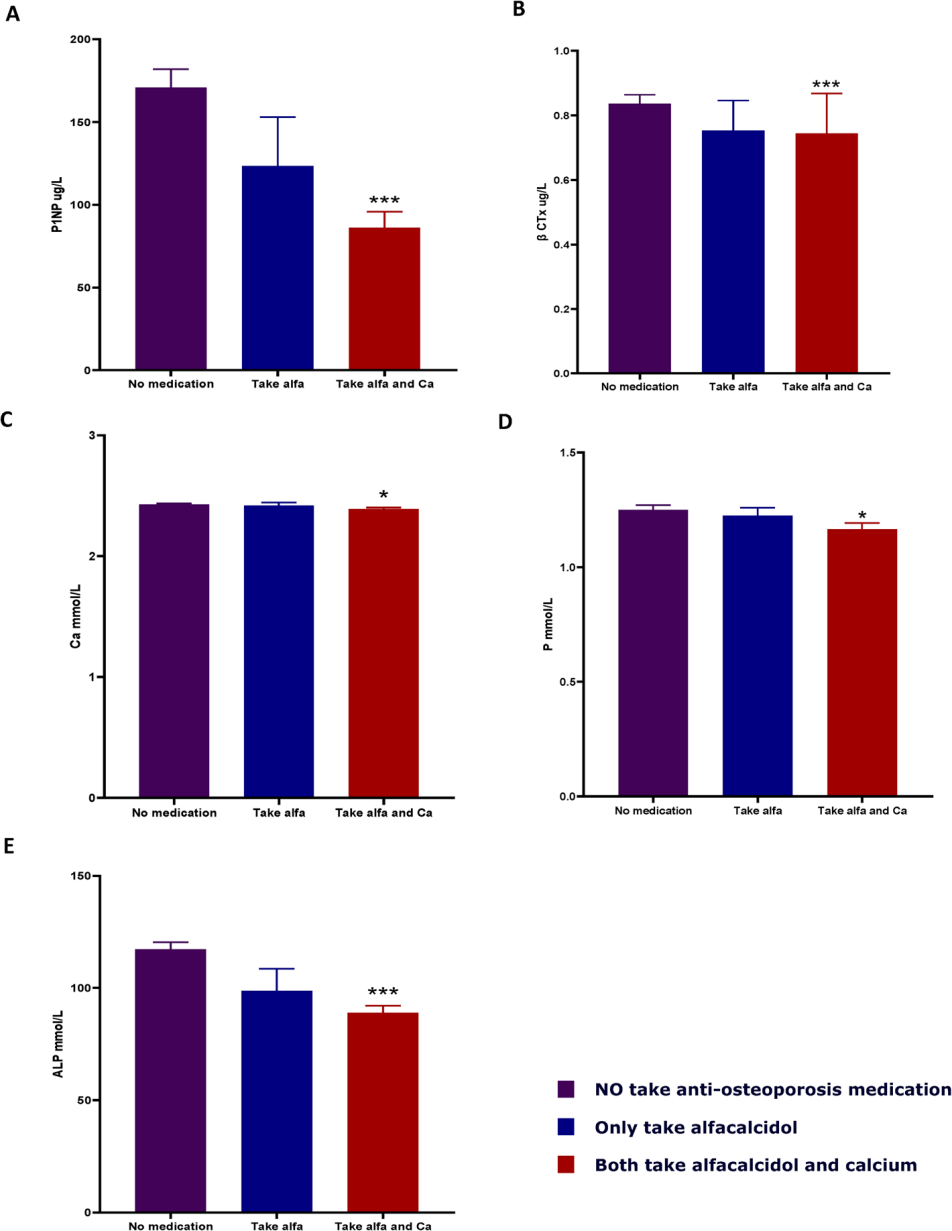
**a** Patients received both alfacalcidol and calcium treatment has a difference than other groups in P1NP; **b** Patients received both alfacalcidol and calcium treatment has a difference than other groups in β-CTx; **c** Patients received both alfacalcidol and calcium treatment has a difference than other groups in Serum Ca; **d** Patients received both alfacalcidol and calcium treatment has a difference than other groups in serum P; **e** Patients received both alfacalcidol and calcium treatment has a difference than other groups in ALP. *p* < 0.05 as assessed by Mann–Whitney U and Kruskal-Wallis tests

Multiple logistic regression analyses found either type of anti-osteoporosis medicines or anti-osteoporosis medicines taking duration time are not the main factors to influence bone turnover and metabolic markers in these patients. However, nonparametric analysis (Bonferroni Correction) found only the group in which patients received both alfacalcidol and calcium treatment has difference than the other groups in P1NP, β-CTx, Serum Ca, P and ALP (Fig. 4a-e).

### Another found

Multiple logistic regression analyses found the 25(OH)D test in summer will get a higher level than other seasons (OR:1.70, 95% CI:1.01–2.60, *p* = 0.044).

## Discussion

The present study focused on bone metabolism indicators in patients of different age and gender using HIS data from one hospital, providing mathematical modeling to assist the future study of age and gender in disease research. Although multiple studies have been published that provide a reference for differences in children and adolescents [2–8], those studies did not provide a precise breakdown of age and gender, namely at what patient age does parameters require precise matching and which age does they not. Researchers believe that age matching should be performed on all subjects in disease control research, but strict age-matching substantially increases the cost and difficulty of conducting the study. The present research found that patients aged more older than 20 years demonstrate that P1NP, β-CTx and 25(OH)D in patient research do not require age matching. This will greatly help researchers reduce the cost and difficulty of their research.

The biological data for young individuals is very complicated, and so we established mathematical models to correlate data for people under the age of 20. We noted that P1NP and β-CTx levels were especially correlated with age. The concentrations of P1NP in infants and young children were higher although their values gradually decreased with age, but not in a simple linear relationship. Firstly, it significantly declined from 0 to 5 years, followed by a period of equilibrium and a small escalation phase between the ages of 6 and 15. Over 15, P1NP levels again rapidly declined by the age of 20 to values close to those of adults. Many studies have demonstrated that P1NP and β-CTx are associated with age, but none have analyzed the relationship in detail or curve fitted the relationship, as conducted here. According to statistical analysis, the relationship with P1NP and age is a complex third-degree polynomial function, and the curve fits well using a cubic relationship. There are two peaks in bone growth in childhood. The first peak appears in infancy and the second in early adolescence. The present study found that bone metabolic indicators grew at their highest rate during puberty, the fastest rate for bone minerals over the age 12–13. As an indicator related to rapid growth rate in healthy individuals, it is not surprising that serum ALP and PINP declines after puberty. β-CTx declined before the age of 4, then increased over the ages 4–14 years, gradually decreasing after the age of 14. In the present study, because R^2^ was less than 0.3, the trend was judged to not be significant, possibly due to the limited sample size. A positive result might have been possible had the sample size been larger. The data obtained from the HIS in the present study included a large number of records of infants and children which allowed us to construct a mathmatical model. Prior to this study, almost no bone metabolic data had been published regarding Chinese children or adolescents, especially babies. In one large study, the minimum age was 15–19 years [9]. Another study that researched a large cohort of healthy adults in China, focused on healthy individuals older than 20 years of age [10]. Thus, studies on children in China, both healthy and sick, are scarce [11, 12]. Studies of pediatric-specific diseases are valuable as reference data. However, due to the sample size and narrow-specificity of the diseases in question, the results are often generally only applicable in a limited fashion [13–15]. Nevertheless, the curve model of young patients obtained in this study provides a good basis for future research.

Bone metabolism is closely related to gender, an association which has been described in multiple studies [9, 16–19]. In the present research, we found significant differences in P1NP, β-CTx, 25(OH)D and ALP concentrations between genders at all ages. However, if study subjects are stratified by age, the differences in gender are not apparent. This suggests that differences in gender depend on the ages of individuals, indicating that gender does not need to be strictly matched if age has been matched. Conversely, if age is not strictly matched, it would be necessary to strictly match by gender. Clearly, in clinical research, it is easier to match gender than age, so matching gender only would be advantageous for future research studies.

The patients who both took alfacalcidol and calcium were analyzed separately. Patients taking both two medication have lower N1NP, β-CTx, 25(OH)D and AL*P* values than others. Taking two kinds of medications did have the correlations to bone turnover and metabolic marker, but we can’t actually judge the causal of the relationship. In bone turnover and metabolic clinical trials, patients who take both alfacalcidol and calcium should be excluded but only take alfacalcidol or calcium didn’t need to be excluded.

## Conclusions

P1NP and β-CTx concentrations are highly correlated with age, especially in individuals under 20 years of age. If these two indicators require analysis in a case-control study, patients and controls must be strictly matched by age or hierarchical research should be conducted according to age if subjects are under 20 years of age. In contrast, researchers do not need to match patients strictly if all subjects are older than 20. The threshold for ALP is 40 years of age. The situation for Ca and P is extremely complicated and we strongly recommend strictly matching according to age in disease research. The difference in P1NP, β-CTx, 25(OH)D and ALP concentrations between genders depends on the difference in age. These don’t require strict gender matching if age has been matched. Medication history should be considered in bone turnover and metabolic clinical research.

## Supplementary information

**Additional file 1: Table S1.** General characteristics of the 36 kinds of diseases.

## Data Availability

The datasets used during the current study are available from the corresponding author on reasonable request.

## Abbreviations

P1NP: Amino-terminal propeptide of type I collagen
β-CTx: Beta-isomerized C-terminal telopeptide of type I collagen
ALP: Alkaline phosphatase
25(OH)D: 25-hydroxyvitamin D
PTH: Parathyroid hormone
Ca: Calcium
P: Phosphorus
