# Supplementary material for: Correlation between bone turnover and metabolic markers with age and gender: a cross-sectional study of hospital information system data
Source: BMC Musculoskelet Disord. 2020 Sep 10;21:603. doi: 10.1186/s12891-020-03610-w (PMC7487991; doi:10.1186/s12891-020-03610-w)
Supplement: Supplementary file 1 — Additional file 1: Table S1. General characteristics of the 36 kinds of diseases. [file 12891_2020_3610_MOESM1_ESM.docx]

Table 1 General characteristics of the 36 kinds of diseases.

| **Number** | **Diagnosis** | **Age** | **Sex** | | **P1NP**  **(μg/L)** | **β-CTx**  **(μg/L)** | **PTH (pmol/L)** | **Ca (mmol/L)** | **P**  **(mmol/L)** | **25(OH)D**  **(μg/L)** | **ALP**  **(mmol/L)** |
| --- | --- | --- | --- | --- | --- | --- | --- | --- | --- | --- | --- |
|  |  |  | **Male**  **(n)** | **Female**  **(n)** |  |  |  |  |  |  |  |
| 1 | Diabetes | 57.00(51.00,65.50) | 100 | 69 | 40.21(27.71, 50.97) | 0.44(0.28,0.64) | 3.05(2.30,4.48) | 2.38(2.29,2.46) | 1.12(1.00,1.22) | 20.70(15.60,26.23) | 74.00(63.00,99.00) |
| 2 | Rheumatoid Arthritis | 52.00(46.00,62.00) | 33 | 94 | 46.58(32.34,77.37) | 0.46(0.29,0.79) | 3.05(2.00,4.07) | 2.41(2.34,2.52) | 1.08(0.98,1.24) | 20.90(24.20,28.80) | 82.00(66.00,98.00) |
| 3 | Osteoarthritis | 59.00(50.50,67.00) | 18 | 79 | 47.66(35.36,69.86) | 0.55(0.31,0.73) | 3.55(2.83,4.76) | 2.42(2.32,2.52) | 1.11(0.97,1.28) | 22.40(18.00,27.60) | 80.00(64.00,96.00) |
| 4 | Systemic Lupus Erythematosus | 33.00(27.00,48.00) | 12 | 81 | 39.71(23.25,76.42) | 0.46(0.30,0.82) | 2.90(1.80,4.10) | 2.40(2.32,2.48) | 1.23(1.05,1.41) | 23.00(18.05,28.10) | 69.00(55.50,83.50) |
| 5 | Ankylosing Spondylitis | 24.00(20.00,33.00) | 18 | 3 | 71.67(56.20,71.67) | 0.76(0.56,1.07) | 3.70(2.55,4.65) | 2.42(2.30,2.58) | 1.15(1.01,1.27) | 21.50(18.90,26.30) | 87.50(70.75,113.00) |
| 6 | Gout | 53.00(40.00,66.00) | 35 | 4 | 44.58(32.51,66.64) | 0.58(0.36,0.84) | 3.80(2.88,5.80) | 2.44(2.32,2.50) | 1.09(0.96,1.25) | 22.20(18.60,28.60) | 89.00(71.00,102.09) |
| 7 | Psoriatic Arthritis | 69.00(62.00,70.00) | 5 | 0 | 37.55(35.11,54.17) | 0.53(0.35,0.63) | 9.70(8.10,9.70) | 2.25(2.22,2.34) | 0.81(0.80,0.81) | 20.70(16.85,29.40) | 60.00(57.00,60.00) |
| 8 | Vasculitis | 39.00(51.00,59.00) | 5 | 10 | 41.25(25.43,60.59) | 0.45(0.30,0.82) | 2.85(1.40,4.75) | 2.39(2.25,2.50) | 1.13(1.05,1.25) | 24.90(21.15,27.30) | 78.00(49.00,104.00) |
| 9 | Myositis Dermatomyositis | 56.00(42.00,61.50) | 2 | 3 | 45.60(28.44,84.43) | 0.74(0.64,0.94) | 2.60(1.40,3.50) | 2.31(2.18,2.50) | 1.31(0.93,1.54) | 19.90(13.00,23.85) | 64.00(45.00,86.50) |
| 10 | Rheumatic Polymyalgia | 54.50(37.50,60.25) | 4 | 6 | 35.45(31.48,43.24) | 0.31(0.25,0.36) | 3.50(2.75,4.48) | 2.44(2.31,2.52) | 1.06(1.00,1.30) | 20.30(15.20,27.20) | 65.00(56.75,80.25) |
| 11 | Cancer | 55.50(51.50,69.00) | 5 | 7 | 63.69(29.54,146.58) | 0.63(0.30,7.95) | 4.60(0.30,7.95) | 2.33(2.21,2.64) | 1.21(0.98,1.47) | 20.20(15.20,25.20) | 90.00(61.50,202.25) |
| 12 | Gastrointestinal Diseases | 46.00(33.00,54.25) | 8 | 8 | 55.75(42.37,66.63) | 0.52(0.41,0.84) | 5.80(3.68,10.68) | 2.42(2.32,2.48) | 1.16(0.94,1.32) | 20.45(17.25,29.00) | 65.00(56.00,92.00) |
| 13 | Sjogren Syndrome | 50.00(42.00,61.50) | 3 | 22 | 40.07(23.82,82.77) | 0.51(0.24,0.77) | 3.40(2.78,4.38) | 2.37(2.32,2.44) | 1.11(1.01,1.23) | 23.00(17.60,26.55) | 65.00(51.00,75.00) |
| 14 | High Blood Pressure Disease | 71.00(61.00,79.00) | 6 | 13 | 34.42(27.29,53.95) | 0.39(0.26,0.50) | 4.60(2.85,6.90) | 2.38(2.31,2.51) | 1.07(1.01,1.13) | 22.70(18.28,25.33) | 71.00(63.00,83.00) |
| 15 | Rickets | 2.34(1.27,6.00) | 12 | 8 | 594.10(457.50,902.10) | 1.26(1.03,1.51) | 3.40(1.83,4.53) | 2.59(2.52,2.66) | 1.56(1.46,1.70) | 31.90(24.00,38.80) | 219.50(202.75,274.00) |
| 16 | Stunt Growth | 8.00(1.83,12.00) | 19 | 23 | 576.20(433.30,828.30) | 1.37(1.13,1.80) | 2.80(1.98,3.85) | 2.57(2.52,2.67) | 1.52(1.41,1.73) | 24.95(19.80,29.88) | 241.50(189.63,284.51) |
| 17 | Bone Fractures | 60.00(40.00,71.00) | 13 | 24 | 56.23(31.00,107.56) | 0.60(0.42,0.86) | 3.30(2.10,4.60) | 1.17(0.95,1.42) | 1.17(0.96,1.42) | 17.40(13.00,22.50) | 96.00(66.64,118.50) |
| 18 | Osteoporosis | 65.00(55.00,88.00) | 6 | 23 | 59.79(39.94,83.32) | 0.62(0.39,0.9) | 3.20(2.30,4.10) | 2.42(2.28,2.48) | 1.12(0.96,1.27) | 20.00(17.50,27.50) | 91.00(70.50,107.50) |
| 19 | Coronary Heart Diseases | 78.00(59.25,80.75) | 2 | 4 | 49.98(39.39,84.12) | 0.62(0.34,0.87) | 5.4(4.45,14.33) | 2.40(2.24,2.69) | 1.01(0.86,1.15) | 16.10(12.86,32.10) | 81.00(59.64,101.00) |
| 20 | Hyperthyroidism | 38.00(28.00,47.00) | 18 | 31 | 157.65(74.22,341.05) | 1.28(0.86,1.65) | 3.00(2.20,5.05) | 2.50(2.39,2.57) | 1.23(1.10,1.42) | 22.60(18.20,28.90) | 105.00(69.00,140.50) |
| 21 | Hypothyroidism | 33.00(28.00,49.00) | 4 | 15 | 51.26(31.02,76.33) | 0.47(0.39,0.76) | 3.30(2.50,5.30) | 2.43(2.33,2.57) | 1.11(0.92,1.37) | 21.70(18.60,28.60) | 73.00(57.00,87.00) |
| 22 | Connective Tissue Diseases | 49.00(31.00,59.00) | 4 | 11 | 55.26(23.07,189.85) | 0.45(0.28,1.25) | 3.00(1.75,3.60) | 2.37(2.27,2.49) | 1.33(1.03,1.41) | 21.95(19.20,23.80) | 103.00(78.00,159.00) |
| 23 | Nephrolithiasis | 52.00(42.00,70.00) | 5 | 6 | 75.08(58.48,296.9) | 0.72(0.51,1.24) | 4.20(3.75,5.23) | 2.42(2.26,2.51) | 1.13(0.87,1.29) | 19.50(16.60,22.40) | 115.00(94.00,274.00) |
| 24 | Chronic Kidney Diseases | 45.00(6.00,55.75) | 7 | 3 | 595.6(210.2,974.08) | 2.74(1.95,3.95) | 11.40(3.90,62.35) | 2.51(2.35,2.94) | 2.05(1.20,2.61) | 20.45(16.50,28.00) | 269.95(63.00,452.00) |
| 25 | Pulmonary Diseases | 27.00(1.84,67.00) | 9 | 4 | 290.6(32.23,912.55) | 0.72(0.37,1.16) | 2.90(1.40,5.28) | 2.35(2.19,2.75) | 1.33(1.18,1.56) | 19.10(14.70,36.65) | 101.00(58.25,358.75) |
| 26 | Brain Diseases | 47.00(11.75,59.00) | 19 | 17 | 71.79(31.58,382.10) | 0.76(0.51,1.32) | 3.00(2.20,3.50) | 2.45(2.34,2.53) | 1.24(1.09,1.49) | 20.90(16.03,29.25) | 88.00(65.75,163.00) |
| 27 | Hyperparathyroidism | 46.00(40.00,49.50) | 1 | 2 | 72.28(70.97,72.28) | 1.08(0.74,1.08) | 25.30(22.50,25.30) | 2.86(2.50,2.86) | 0.58(0.57,0.58) | 18.20(12.20,18.20) | 113.67(88.00,113.67) |
| 28 | Normal Examination | 9.00(1.75,13.00) | 9 | 8 | 481.40(315.8,824.6) | 1.13(0.68,1.56) | 3.20(2.35,4.25) | 2.58(2.45,2.69) | 1.45(1.28,1.74) | 24.06(20.00,30.09) | 250.00(144.50,297.50) |
| 29 | Adrenal Tumors | 59.00(45.00,65.50) | 7 | 6 | 36.72(32.13,68.75) | 0.41(0.28,0.57) | 4.60(3.50,4.60) | 2.37(2.32,2.43) | 1.09(0.94,1.13) | 21.40(18.25,26.05) | 68.92(46.00,78.50) |
| 30 | Kidney Transplantation | 41.00(31.00,43.50) | 7 | 2 | 427.3(42.40,496.5) | 2.66(0.90,4.24) | 11.45(5.00,31.15) | 2.59(2.10,2.81) | 0.81(0.55,0.99) | 19.60(16.85,27.35) | 154.00(124.09,220.00) |
| 31 | Fibromyalgia | 52.50(41.75,58.00) | 1 | 5 | 42.99(36.25,49.68) | 0.50(0.37,0.61) | 3.80(3.35,4.50) | 2.41(2.16,2.46) | 1.23(1.06,1.27) | 22.70(20.35,27.48) | 66.50(60.25,77.50) |
| 32 | Systemic Scleroderma | 54.00(38.00,66.50) | 0 | 9 | 37.90(29.75,54.17) | 0.50(0.43,0.74) | 3.90(2.68,5.03) | 2.40(2.28,2.46) | 1.14(1.1,1.32) | 17.5(11.15,21.80) | 56.00(48.50,72.00) |
| 33 | Hypocalcemia | 34.00(31.00,57.25) | 3 | 1 | 34.10(23.41,58.43) | 0.15(0.13,0.18) | 0.30(0.05,0.85) | 1.86(1.30,2.51) | 2.14(1.51,2.23) | 21.20(18.88,28.10) | 52.50(46.00,69.50) |
| 34 | Hypophosphatemia | 40.00(39.00,41.00) | 11 | 1 | 44.00(37.22,60.27) | 0.67(0.48,0.77) | 26.85(20.38,32.33) | 2.28(2.23,2.40) | 0.71(0.62,0.77) | 20.10(19.30,27.30) | 116.00(96.00,135.75) |
| 35 | Blood Disorders | 27.00(13.29,64.50) | 2 | 3 | 67.34(44.62,216.45) | 0.78(0.44,1.79) | 1.50(1.30,4.20) | 2.42(2.35,2.52) | 1.25(1.07,1.95) | 19(15.15,21.95) | 159.00(98.00,192.00) |
| 36 | others | 22.50(11.00,58.25) | 9 | 9 | 89.03(42.87,520.45) | 1.02(0.51,2.60) | 4.60(2.40,5.70) | 2.46(2.26,2.49) | 1.47(1.11,1.66) | 20.3(15.65,23.73) | 108.00(71.00,245.00) |

Data are presented as Median (QR). P1NP: Propeptide of type I collagen; β-CTx: Beta C-terminal cross-linked telopeptides of type I collagen; PTH: Parathyroid hormone ; Ca: Calcium; P: Phosphorus; 25(OH)D: 25-hydroxyvitamin D; ALP: Alkaline phosphatase.
